# Parental Internet-Specific Rules and the Onset of Adolescents’ Problematic Social Media Use: Prospective Study Testing Potential Moderators

**DOI:** 10.2196/64252

**Published:** 2025-09-18

**Authors:** Suzanne Geurts, Ina Koning, Regina Van den Eijnden, Helen Vossen

**Affiliations:** 1 Utrecht University, Padualaan 14, Utrecht, 3584CH, The Netherlands, 31 623807447; 2 Vrije Universiteit Amsterdam, Amsterdam, The Netherlands

**Keywords:** adolescents' problematic social media use, restrictive mediation, preventive effect, moderators, adolescent, parents, parenting, parental phubbing, social media, problematic internet use, addictive, addiction, parental mediation, health informatics, eHealth

## Abstract

**Background:**

Many parents are concerned about their adolescents’ (problematic) social media use. Therefore, parents may apply restrictive mediation practices to prevent problematic social media use. However, their effectiveness remains unclear.

**Objective:**

This study aimed to provide insights into the specific groups and conditions under which restrictive mediation may effectively prevent adolescents’ problematic social media use. Specifically, we investigated the prospective relationship between rules about the amount, location, and timing of internet use and the onset of adolescents’ at-risk or problematic social media use. In addition, we examined the moderating role of demographic and parenting factors, including adolescents’ age, adolescents’ gender, adolescent involvement in rule-setting, positive parenting, parental phubbing, and quality of coparenting (2-way interactions). Furthermore, we explored whether the moderation effects of the parenting factors varied by adolescents’ age and gender (3-way interactions).

**Methods:**

Four-wave survey data of 315 adolescents (T1: mean age 13.44, SD 2.26 years; n=146, 46.3% girls, n=169, 53.7% boys) and their parents (T1: mean age 46.4, SD 5.05 years; n=292, 55.4% mothers) were used. Data were collected between April 2020 and January 2022, with a 6-month interval between each wave.

**Results:**

No significant main effect was found of internet-specific rule-setting at T1 on the onset of adolescents’ at-risk or problematic social media use throughout the study period (odds ratio [OR] 0.959, 95% CI 0.60-1.54, *d*=0.02). Yet, 2-way interaction analyses revealed that the effect of internet-specific rule-setting varied by adolescents’ age (OR 2.171, 95% CI [1.35-3.49], *d*=0.43). Specifically, for adolescents aged <12.31 years (−0.5 SD), stricter rules were associated with a lower likelihood of developing at-risk or problematic social media use (unstandardized beta (B)=−0.568, SE=0.280, 95% CI [–1.12 to –0.02], *P*=.042). In contrast, for adolescents aged >15.70 years (+1 SD), stricter rules were associated with a higher likelihood of developing at-risk or problematic social media use (B=0.594, SE=0.294, 95% CI 0.02-1.17, *P*=.043). Two-way interaction effects of rule-setting with adolescents’ gender (OR 0.945, 95% CI 0.54-1.64, *d*=0.03), adolescent involvement in rule-setting (OR 1.02, 95% CI 0.77-1.36, *d*=0.01), positive parenting (OR 1.044, 95% CI 0.69-1.59, *d*=0.02), parental phubbing (OR 0.977, 95% CI [0.72-1.33], *d*=0.01), and quality of coparenting (OR 0.877, 95% CI 0.64-1.21, *d*=0.07) were not significant, nor were any of the 3-way interaction effects.

**Conclusions:**

Setting internet-specific rules seems to have a preventive effect on the development of problematic social media use symptoms in pre- and early adolescence, but may be counterproductive from the age of 15.7 years onward. These findings highlight the importance of age-appropriate parental mediation strategies to prevent problematic social media use.

## Introduction

### Background

Out of concern for problematic, addictive-like use of social media, many parents limit adolescents’ internet use by regulating the time spent online or the access to certain platforms, that is, restrictive mediation [1]. Although restrictive mediation has appeared effective in reducing the amount of time adolescents spend online [2], the effect on problematic internet use remains unclear. Previous reviews and meta-analyses have shown inconsistent associations [3-5], highlighting the need to examine potential moderators that might influence this relationship. Moreover, almost no longitudinal studies have been conducted, and the few longitudinal studies that do exist have failed to disentangle preventive effects from “intervention” effects. Therefore, this study examines the prospective relationship between rules about amount, location, and timing of internet use and the onset of adolescents’ problematic social media use symptoms, specifically testing the preventive effect of parental internet-specific rule-setting. In addition, we investigate several demographic and parenting factors as potential moderators.

### Internet-Specific Rules and Problematic Social Media Use

Problematic social media use refers to a behavioral pattern in which individuals experience negative consequences and difficulties in different aspects of daily life due to compulsive engagement with social media platforms [6]. Problematic social media use is commonly assessed based on the presence of addiction symptoms such as loss of control and withdrawal symptoms when not online. Using the Social Media Disorder (SMD) Scale [7], researchers classify participants as problematic social media users when they endorse at least 6 of the 9 symptoms [8]. According to this criterion, prevalence rates range from 3.2% to 16.4% across different countries [9]. However, a substantially higher percentage of adolescents shows between 2-5 symptoms (eg, 34.7% of Dutch adolescents) [8]. These adolescents can be considered at risk for problematic social media use and, similar to problematic users [10,11], are at an increased risk for various negative outcomes related to physical, social, and mental well-being [8]. Consequently, preventing normative users from becoming at-risk and problematic users is crucial to safeguard their well-being.

Parents are assumed to play a critical role in fostering healthy media habits, as they are pivotal socialization agents and continue to be the primary gatekeepers of adolescents’ access to media devices and content [12]. They use various strategies and approaches to manage, guide, and control their children’s internet use, among which restrictive mediation is common. Restrictive mediation refers to setting boundaries and limitations [13]. This is a commonly used parenting strategy, particularly when concerned about the risks and adverse consequences of media use, such as the development of problematic social media use [14,15].

The effectiveness of restrictive mediation in preventing problematic social media use has, however, not been established. Previous studies presented inconclusive findings and predominantly relied on cross-sectional designs [4,5]. In addition, most previous studies on the association between restrictive mediation and problematic internet use used measures of restrictive mediation from 2010 or earlier, primarily concentrating on restrictions on what children do online, including sharing personal information, photos, and video, downloading music and films, and buying online [16,17]. However, rules regarding how long, where, and when adolescents are allowed to use the internet seem more relevant to problematic social media use, as it is generally assumed that spending too much time online is an important risk factor for, and indicator of problematic social media use [7].

A few studies used longitudinal data to test the association between rules about the amount, location, and timing of internet use and problematic social media use. One of them revealed a (marginally) significant protective effect of these rules on problematic social media use for girls [18]. Another study showed that a perceived increase in such rules was not related to a change in problematic social media use symptoms among adolescents one year later [19]. Rather, an increase in problematic social media use symptoms resulted in a decrease in internet-specific rules over time. However, these studies did not distinguish between preventive and intervention effects. This is important because parental rules may be more likely to prevent rather than reduce adolescents’ problematic social media use. Clear boundaries and expectations regarding online behavior shape adolescents’ attitudes and habits. When these rules are effectively enforced, adolescents are less likely to develop problematic patterns of social media use in the first place, thereby preventing the onset of problematic social media use. However, if problematic social media use has already developed, parental rules may have limited efficacy in reducing its symptoms, as they primarily address behavior rather than underlying addictive tendencies and/or psychological factors (eg, unsatisfied psychological needs such as the need for relatedness) [20]. Similarly, research on adolescents’ alcohol use has shown that parental rules have a smaller effect on reducing alcohol use once adolescents have started drinking but have a greater effect on preventing or delaying the onset of alcohol use [21,22]. Therefore, we will explicitly examine the preventive effect of rules about the amount, timing, and location of internet use by testing whether these rules prospectively predict the onset of at-risk and problematic social media use.

### Moderating Factors

The preventive effect of parental internet-specific rules may depend on for whom they are set, how they are set, and the conditions under which these rules are applied. Therefore, this study will take into account important demographic and parenting moderators. As demographic moderators, adolescents’ age and gender will be considered. Due to their growing desire for autonomy [23], compliance with family rules is likely to decline as adolescents age [24]. This increased desire for autonomy may lead older adolescents to experience parental rules as less legitimate, making them more inclined to reject these rules [25]. In line with this, a recent meta-analysis showed that restrictive mediation was negatively related to problematic media use among children, but not among adolescents [3]. Another meta-analysis showed that restrictive mediation was positively related to problematic internet use among older adolescents [4]. In addition, previous research has shown that girls are more compliant and responsive to parental rules than boys [26]. Moreover, one study that examined the effect of internet-specific rule-setting on problematic social media use over time separately for boys and girls found only a (marginally) significant effect among girls [18]. Therefore, we expect the protective effect of internet-specific rule-setting to be particularly pronounced among younger adolescents and girls.

Adolescents’ involvement in internet-specific rule-setting, positive parenting, parental phubbing, and quality of internet-specific coparenting will be considered as parenting moderators. The self-determination theory [27] proposes that socialization practices are more successful when applied in an autonomy-supportive way, which is supported by empirical evidence [21,28]. Following this theory, having internet-specific rules may be more effective in preventing problematic social media use when these rules are set together with the adolescent rather than solely imposed by parents. By establishing the rules collaboratively, the perspective of the adolescent is considered and the rationale behind the rules is made clear, which gives adolescents a sense of autonomy and fairness [29]. Consequently, adolescents may be more likely to adhere to and internalize these rules, leading to more effective regulation of their social media use.

As argued in the integrative model of Darling and Steinberg [30], the broader general parenting style may also influence the effect of parenting practices on children’s behavior. While adolescent involvement in internet-specific rules-setting indicates an autonomy-supportive way of internet-specific parenting, the general parenting style describes the majority of parent-child interactions across a wide range of domains [30]. A positive parenting style is marked by demonstrating love, affection, and autonomy support along with setting clear boundaries and may enhance openness to socialization, which in turn may increase the effectiveness of rule-setting. A recent longitudinal study, indeed, found that limiting adolescents’ internet use in combination with an autonomy-supportive, warm parenting style predicted fewer problematic social media use symptoms over time than limiting adolescents’ internet use in combination with a less autonomy-supportive parenting style [31]. Although parents with a more positive general parenting style are more likely to incorporate adolescents’ perspective into rule-setting regarding internet use [32], this does not necessarily have to be the case [33]. For example, some parents may have a positive parenting style across many domains, but they might be very strict in the domain of internet use and set rules without involving their child’s opinion. Therefore, we also include positive parenting in general as a moderator.

Another important contextual factor to consider is parental phubbing. Parental phubbing refers to the extent to which parents use their smartphone during interactions with their child [34]. Fu et al [35] showed that the association between parental active mediation and adolescents’ positive behavioral attitudes toward self-regulating their mobile phone usage was weaker for adolescents who perceived higher levels of parental phubbing. Moreover, findings from Liu et al [36] suggested that parents’ actual internet use has a greater influence on adolescents’ problematic internet use than the communicated norms when there is inconsistency between the parents’ behavior and their stated norms. These findings suggest that parental phubbing may diminish the effectiveness of rules.

Finally, internet-specific coparenting will be considered as a parenting moderator. Coparenting encompasses how parents coordinate, interact, and support each other in shared parenting tasks [37]. It is crucial to acknowledge coparenting processes when examining the effect of restrictive mediation, as parents might differ in their media attitudes and rules [38-41]. When there are disparities in enforcing rules between both parents, parental rules tend to be less effective in regulating children’s and adolescents’ behavior [42,43], as parents may disregard or undermine the other’s parenting practices. Furthermore, when adolescents receive mixed messages, it is unclear to them which guidelines to follow [43]. In turn, they may be less likely to internalize these rules.

### Objective

In this study, we investigate the prospective relationship between rules about the amount, location, and timing of internet use and the onset of adolescents’ at-risk or problematic social media use, and the moderating role of two demographic (adolescents’ age and gender) and four parenting factors (adolescent involvement in rule-setting, positive parenting, parental phubbing, and quality of coparenting). We hypothesize that adolescents who perceive more parental internet-specific rule-setting are less likely to develop at-risk or problematic social media use over the course of the study (H1). Regarding the demographic moderators, we hypothesize that the preventive effect of internet-specific rule-setting will be particularly pronounced among younger adolescents (H2.a) and among girls (H2.b). Regarding the parenting factors, we hypothesize that internet-specific rules are more likely to be effective in preventing at-risk and problematic social media use when adolescents are more involved in rule-setting by their parents (H.3a), when adolescents perceive more positive general parenting (H.3b), when adolescents experience less parental phubbing (H.3c), and when parents report better coparenting (H3.d). In addition, we will test whether the moderation effects of the parenting factors depend on adolescents’ gender and age. These 3-way interaction effects will be examined exploratorily.

## Methods

### Procedure

Data from this study (adolescent and parent data) were collected within the Digital Family project, which is a longitudinal Dutch research project on youth digital media use and the family context [31]. Families were recruited using convenience sampling techniques. Recruitment advertisements were distributed through schools and sports clubs, door-to-door flyer distribution in 6 different places across the Netherlands (both urban and rural areas), social media platforms including Facebook and LinkedIn, advertisements on a website for parents (ouders.nl), as well as word of mouth. Data collection was conducted via closed online surveys administered through Qualtrics, requiring participants to log in with a unique participant number and password. Criteria for inclusion were Dutch-speaking families with children between the ages of 10 and 18 years, preferably participating with at least one parent or caregiver (in this paper referred to as “parent”) with a maximum of 2 parents and 2 children. Certain items in the survey were conditionally displayed based on the responses to other items to reduce the number of questions. In addition, a completeness check was applied. Participants were not able to review and change their answers in previous parts to prevent possible influences of questions asked later in the survey. The survey was tested by the authors and a 10-year-old adolescent before the study. We used the unique participant number and password to manage duplicate entries. In cases of duplicates, we retained the most complete entry or, if entries were equally complete, the first one. The data include 4 waves with approximately 6 months in between (T1: April-July 2020; T2: November-December 2020; T3: May-July 2021; T4: November 2021-January 2022). In each subsequent wave, new families were invited to participate alongside those from previous waves. We followed the Checklist for Reporting Results of Internet E-Surveys (CHERRIES) in reporting the study procedure.

### Participants

In total, 403 adolescents and 398 parents participated in the first wave of the Digital Family project, 386 adolescents and 414 parents in the second wave, 260 adolescents and 287 parents in the third wave, and 241 adolescents and 267 parents in the fourth wave. Since participation took place at home without supervision by a researcher and participants received an incentive for completing the questionnaire, we screened the data for careless responding. Participants were excluded if they showed invariable or contradictory responses, or if they failed the attention check item. We removed 14 adolescents and 11 parents at T1, 7 adolescents and 10 parents at T2, 18 adolescents and 21 parents at T3, and 17 adolescents and 4 parents at T4. For participants recruited at later waves (adolescents: n=121 at T2, n=36 at T3, and n=9 at T4; parent 1: n=104 at T2, n=29 at T3, and n=7 at T4; parent 2: n=186 at T2, n=9 at T3, and n=0 at T4), their first participating wave was treated as equivalent to T1 to increase the sample size. Participants recruited at later waves did not significantly differ on study variables from participants who participated from the first wave. As we aimed to capture the onset of at-risk and problematic social media use (ie, a transition from normative to at-risk or problematic social media use), we selected adolescents who scored zero or one symptom of problematic social media use (indicating normative use) at T1, along with their parents. This resulted in an analysis sample of 315 cases (adolescents and one or two caregivers, in this paper referred to as parents), participating at all 4 waves, aside from attrition over time, which is detailed in the “Results” section. The adolescents were aged 9‐19 years (mean 13.44, SD 2.26), of which 46.3% (n=146) were girls and 53.7% (n=169) were boys. Of the participants, 97.7% (n=304) were born in the Netherlands. The remaining participants were born in Belgium (n=1), Syria (n=2), Australia (n=2), Pakistan (n=1), and Scotland (n=1). For 4 participants, information on their country of birth was missing. The majority were in secondary school (n=200, 63.5%), with 24.5% (n=49) of them attending prevocational education (VMBO or VMBO/HAVO), 28.5% (n=57) general higher education (HAVO or HAVO/VWO), and 47% (n=94) preuniversity education (VWO). Of the remaining adolescents, 29.5% (n=93) were in primary school, 4.4% (n=14) in secondary vocational education (MBO), and 1.9% (n=6) in higher professional education (HBO) or university (WO). Of the participating parents (n=529), 55.4% (n=293) were female, and 44.6% (n=236) were male. The mean age of parents was 46.45 years (SD 5.05). Most of the parents (95.4%, n=501) were born in the Netherlands. Other countries of birth included Syria (n=4), Papua New Guinea (n=3), Germany (n=2), Canada (n=2), Peru (n=2), Russia, Pakistan, and Colombia. Parental education level was as follows: 71% (n=374) had completed college or university, 19.3% (n=51) had completed vocational education (MBO), 19.8% (n=47) had completed secondary school, and 0.3% (n=1) had completed primary school.

### Measures

#### Onset of Adolescents’ At-Risk and Problematic Social Media Use

Adolescents’ onset of problematic social media use was assessed through self-reports using the Social Media Disorder Scale [7], consisting of nine items corresponding to the nine addiction symptoms: preoccupation, persistence, tolerance, withdrawal, displacement, problems, escape, deception, and conflict. Participants responded to each item with yes (1) or no (0), stating whether they exhibited the described behavior in the past year. For example: “During the past year, have you regularly found that you cannot think of anything else but the moment that you will be able to use social media again?” The total number of symptoms present was calculated by summing the item scores. Subsequently, these sum scores were recoded into a dichotomous variable reflecting normative (0‐1 symptom; coded as 0) versus at-risk or problematic use (1+ symptoms; coded as 1). These cut-off scores for classification are based on before latent class analyses on the 9 SMD scale items in a nationally representative sample of Dutch adolescents. This research identified three distinct groups of users: normative users (≤1 symptom), at-risk users (2‐5 symptoms), and problematic users (≥6 symptoms) [8]. Next, the scores on this variable at T2, T3, and T4 were summed and recoded. Participants were coded as 0 if they reported normative use (score of 0) at all 3 waves and coded as 1 if they reported at-risk or problematic use (score of ≥1) at any of these 3 waves. As only participants who reported normative use at T1 were included, this variable indicates whether participants shifted from normative use to at-risk or problematic social media use during the course of the study (0=no onset of at-risk or problematic social media use, 1=onset of at-risk or problematic social media use). Tetrachoric ordinal α was 0.86 at T1, 0.84 at T2, 0.90 at T3, and 0.81 at T4.

#### Internet-Specific Rule-Setting

Internet-specific rule-setting at T1 was measured with 5 items derived from Koning et al [18] and 3 additional items [32]. Adolescents rated on a 5-point Likert scale (1=never, 5=very often) to what extent they were allowed to “use the internet or play games as long as they wanted,” “use the internet or play games for more than three hours,” “use the internet or play games while their homework was not finished yet,” “use the internet or play games in the hour before going to sleep,” “bring their smartphone or tablet to their bedroom when going to sleep at night,” “keep their smartphone or tablet with them while doing homework,” “keep their smartphone or tablet with them during dinner,” and “keep on using their smartphone or tablet while talking with their parents” in the past 2 weeks. Reversed scores on the items were combined into a mean score, with higher scores reflecting stricter internet-specific rule-setting. Cronbach α was 0.82.

#### Adolescents’ Involvement in Internet-Specific Rule-Setting

Adolescents’ involvement in internet-specific rule-setting at T1 was assessed by asking parents: “To what extent do you involve your children’s view in setting up rules regarding media use?” [32]. The item was answered on a 5-point Likert scale ranging from 1 (never) to 5 (always). A mean score of both parents was used when data of 2 parents were available. Higher scores represented greater adolescent involvement in internet-specific rule-setting. The intraclass correlation was 0.45, indicating poor agreement between parents.

#### Positive Parenting

Positive parenting at T1 was measured using 11 items from the Parenting Style Inventory II [44], assessing the three core dimensions of positive parenting: responsiveness (four items: “I can count on my parents or caregivers to help me out if I have a problem,” “My parents or caregivers hardly ever praise me for doing well,” “My parents or caregivers and I do things that are fun together”), demandingness (“My parents or caregivers expect me to follow family rules,” “My parents or caregivers let me get away with things,” “If I don’t behave myself, my parents or caregivers will punish me,” “My parents or caregivers point out ways I could do better”) and autonomy-granting (“My parents or caregivers respect my privacy,” “My parents or caregivers give me a lot of freedom,” “My parents or caregivers make most of the decisions about what I can do,” “My parents or caregivers believe I have a right to my own point of view”). One item of the demandingness subscale was removed (“If I do not behave myself, my parents or caregivers will punish me”) to enhance internal validity (Cronbach α increased from 0.64 to 0.70). Adolescents answered these items on a 5-point Likert scale (1=totally disagree, 5=totally agree). A mean score across the 10 items was calculated, with some items reversed-coded to ensure that higher scores indicated higher levels of positive parenting. Cronbach α was 0.63

#### Parental Phubbing

Parental phubbing at T1 was assessed through 3 items asking adolescents about the frequency of their parents’ smartphone use in three different situations over the past 2 weeks: during dinner together, while talking to each other, and while engaging in fun activities together [32]. Answer categories ranged from 1 (never) to 5 (very often). Mean scores were computed, with higher scores indicating more parental phubbing. Cronbach α was 0.65.

#### Quality of Internet-Specific Coparenting

Quality of internet-specific coparenting at T1 was assessed with 3 adapted items from the VG&O (Vragenlijsten Gezin & Opvoeding) questionnaire, developed in Dutch youth care to measure various parenting aspects [45]. The items were slightly adapted to assess the quality of coparenting specifically in the context of media parenting. Parents reported on a 4-point Likert scale (1=does not apply, 4=applies completely) to what extent the following three statements applied to them: “I feel supported by my partner in the media parenting of our child(ren),” “I can talk with my partner about the media parenting of our child(ren),” and “My partner and I are usually on the same page regarding the media parenting of our child(ren).” Items were combined into a mean score, with higher scores representing better internet-specific coparenting. Where both parents participated, the scores of both parents were averaged. The intraclass correlation was 0.59, indicating moderate agreement between parents. Cronbach α was 0.88.

#### Adolescents’ Gender

For adolescents’ gender, we asked adolescents: “Are you a boy or a girl?” at T1. The response option “*boy*” was coded 0, and the response option “*girl*” was coded 1.

#### Adolescents’ Age

Adolescents’ age was calculated by subtracting the date of birth from their date of participation at T1.

### Data Analysis

#### Descriptives and Bivariate Analysis

Descriptives and correlations were computed in IBM SPSS version 28 (IBM Corp.). Spearman rho was used for correlations with gender and at-risk or problematic social media use. Pearson correlation was used for correlations between all other variables.

#### Multivariate Analysis

To answer the research questions, logistic regression analyses were conducted in Mplus version 8 (Muthén & Muthén) [46]. Full-information maximum likelihood estimation was used to handle missing data, and maximum likelihood estimation with robust SEs was used to adjust for the clustered data (adolescents were clustered within families). In the first model (Model 1), we specified the main effect of internet-specific rule setting on the onset of adolescents’ at-risk or problematic social media use. Adolescents’ age and gender were included as covariates. Next, we examined 2-way interaction effects of adolescent involvement in rule setting, positive parenting, parental phubbing, quality of coparenting, adolescents’ age, and adolescents’ gender with internet-specific rule setting, each separately in its own model (Models 2a-2f). To do so, the particular moderator and its interaction term with internet-specific rule setting were added to the first model. Subsequently, to test whether the moderation effects of adolescent involvement in rule setting, positive parenting, parental phubbing, and quality of coparenting depended on adolescents’ age or gender, the particular 3-way interaction term was added (ie, internet-specific rule-setting*positive parenting*age or internet-specific rule-setting*positive parenting*gender; Models 3a-3h). To avoid multicollinearity, the predictor and moderators were standardized. For significant interaction effects, the Johnson-Neyman technique was used to determine the precise regions of significance [47]. The Benjamini-Hochberg procedure was used to correct the significance level to manage the false discovery rate for the 15 analyses [48]. We applied a false discovery rate of 5%. Cohen *d* was calculated as a measure of effect size. Effect sizes of *d*≥0.20, *d*≥0.50, and *d*≥0.80 were considered small, medium, and large, respectively [49]. The analyses were preregistered in Open Science Framework [50].

### Ethical Considerations

The Ethics Committee of the Faculty of Social and Behavioral Sciences at Utrecht University approved the study protocol (FETC20-192). Participants filled the questionnaires individually and voluntarily at home after giving active informed consent at the start of the survey. In addition to adolescents’ own consent, their parents also provided active parental consent for their participation through the registration form, where they were informed about the study’s purpose, survey duration, data storage and retention, and their rights. This information was reiterated at the beginning of each questionnaire. Data were pseudonymized by removing personal identifiers from the data files. The personal data linked to the participant numbers were stored in a separate file, accessible only to project members. Participants were rewarded with a gift card of €5 (US $5.40) for each completed wave, and at the end of each wave, several gift cards of €100 were raffled among families.

## Results

### Descriptives and Bivariate Analysis

Means, SDs, and correlations are shown in Table 1. Of the sample, 33.5% (n=65) shifted from normative to at-risk or problematic social media use during the course of the study. Mean scores on internet-specific rule-setting, adolescent involvement in rule-setting, positive parenting, and quality of coparenting were relatively high, while the mean score on phubbing was relatively low. Correlations showed that being older was related to less strict internet-specific rule-setting and to more frequent involvement in rule-setting. Being a girl was related to more positive parenting. Moreover, parental phubbing correlated negatively with internet-specific rule-setting, positive parenting, and quality of coparenting, indicating that parents who phubbed more frequently set less strict rules about internet use, were less involved in positive parenting, and reported poorer quality of internet-specific coparenting. In addition, more internet-specific rule-setting was correlated with more adolescent involvement in rule-setting. Finally, parents who reported poorer quality of coparenting reported less frequent involvement of their children in rule-setting and less positive parenting. As expected, there were missing values on the outcome variable, the onset of at-risk or problematic social media use (38.4%, n=121), due to drop-out after T1. Boys (odds ratio [OR] = 0.470, 95% CI 0.29-0.75; *P*=.002), older adolescents (OR= 1.118, 95% CI 1.01-1.24; *P*=.032), and adolescents who experienced less strict internet-specific rule-setting (OR 0.687, 95% CI 0.53-0.89; *P*=.004), less positive parenting (OR 0.514, 95% CI 0.28-0.96; *P*=.035) and more adolescent involvement in rule-setting (OR 1.508, 95% CI 1.06-2.14; *P*=.021) were more likely to drop out after T1.

**Table 1.** Descriptives and correlations between all study variables.

Variable	Value	Range	1	2	3	4	5	6	7
Adolescents’ age, mean (SD)	13.44 (2.26)	9‐19							
*r^a^/ρ^b^*			1.00	—	—	—	—	—	—
*P* value			—	—	—	—	—	—	—
Adolescents’ gender^c^, %	53.7	—							
*ρ*			−0.02	1.00	—	—	—	—	—
*P* value			.78	—	—	—	—	—	—
Rule-setting, mean (SD)	3.61 (0.89)	1‐5							
*r / ρ*			−0.66	−0.04	1.00	—	—	—	—
*P* value			.000	.46	—	—	—	—	—
Adolescents’ involvement in rule-setting, mean (SD)	3.56 (0.69)	1‐5							
*r / ρ*			0.16	−0.02	−0.12	1.00	—	—	—
*P* value			.006	.79	.04	—	—	—	—
Positive parenting, mean (SD)	4.12 (0.37)	1‐5							
*r / ρ*			−0.06	0.12	0.02	0.01	1.00	—	—
*P* value			.28	.04	.68	.93	—	—	—
Phubbing, mean (SD)	1.75 (0.66)	1‐5							
*r / ρ*			0.06	−0.04	−0.19	−0.04	−0.17	1.00	—
*P* value			.32	.52	.000	.48	.003	—	—
Quality of coparenting, mean (SD)	3.35 (0.57)	1‐4							
*r / ρ*			−0.01	0.02	−0.01	0.33	0.13	−0.12	1.00
*P* value			.93	.72	.94	.000	.000	.04	—
Onset at-risk/problematic social media use^d^^,^ %	66.5%	—							
*ρ*			−0.12	0.08	0.03	0.01	−0.04	0.03	−0.07
*P* value			.10	.29	.73	.87	.55	.65	.37

a Pearson correlation (*r*) was used for correlations between all other variables.

b Spearman rho (*ρ*) was used for correlations with gender and at-risk or problematic social media use.

c Reference category: boy.

d Reference category: no onset of at-risk or problematic social media use.

### Multivariate Analysis

#### Main Effect of Internet-Specific Rule-Setting on the Onset of Adolescents’ At-Risk or Problematic Social Media Use

Model 1 tested the main effect of internet-specific rule-setting at T1 on the onset of adolescents’ at-risk or problematic social media use during the study, while controlling for adolescents’ age and gender. Contrary to expectations, internet-specific rule-setting did not significantly predict the onset of adolescents’ at-risk or problematic social media use (Table 2).

**Table 2.** Main effect of internet-specific rule-setting on the onset of at-risk/problematic social media use.

Variable	Model 1	
	OR^a^ (95% CI)	*d* ^b^
Adolescents’ age	0.884 (0.73-1.08)	0.07
Adolescents’ gender^c^	1.393 (0.74-2.63)	0.18
Rule-setting	0.959 (0.60-1.54)	0.02

a OR: odds ratio.

b *d*: Cohen *d.*

c Reference category: boy.

#### Two-Way Interactions

**Figure 1.**
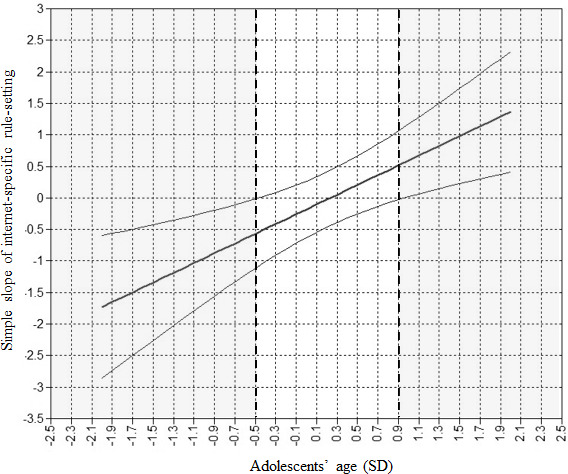
Johnson-Neyman plot of the interaction effect of internet-specific rule-setting and adolescents’ age in the prediction of the onset of at-risk or problematic social media use. The thick line in the center represents the point estimates of the effect of internet-specific rule-setting on the onset of at-risk or problematic social media use at different ages of the adolescent (displayed in SDs). The thinner lines represent the upper and lower limits of the 95% CIs. For values of adolescents’ age where the 95% CIs do not include zero, the effect of internet-specific rule-setting on the onset of at-risk or problematic social media use is significant (gray areas).

In Models 2a-2f, the 2-way interaction term of internet-specific rule-setting with either adolescent involvement in rule-setting, positive parenting, parental phubbing, adolescents’ age, or adolescents’ gender, along with the main effect of the particular moderator, were added. Unexpectedly, none of the variables moderated the association between internet-specific rule-setting and the onset of at-risk or problematic social media use, except for adolescents’ age (Tables3^5^). The Johnson-Neyman approach revealed that among adolescents younger than 12.31 years (−0.5 *SD*), internet-specific rule-setting negatively predicted the onset of at-risk or problematic social media use (B=−0.568, SE=0.28, 95% CI [-1.12, -0.02], *P*=.042). That is, among this age group, adolescents were less likely to develop at-risk and problematic social media use later in time when they reported more strict parental rules regarding internet use at T1. This effect was found to further strengthen as adolescents’ age decreased. Among adolescents 15.70 years or older (+1 SD), internet-specific rule-setting positively predicted the onset of at-risk and problematic social media use (B=0.594, SE=0.294, 95% CI 0.02-1.17; *P*=.043). That is, among this age group, adolescents were more likely to develop at-risk and problematic social media use later in time when they reported more strict parental rules regarding internet use at T1. The effect became stronger as adolescents aged. Among adolescents between the ages of 12.31 and 15.70 years, internet-specific rule-setting did not significantly predict the onset of at-risk or problematic social media use (Figure 1).

**Table 3.** Results of two-way interaction effects with internet-specific rule-setting in predicting the onset of at-risk or problematic social media use.

Variable	Model 2a		Model 2b	
	OR^a^ (95% CI)	*d^c^*	OR (95% CI)	*d*
Adolescents’ age	0.881 (0.72-1.07)	0.07	0.875 (0.72-1.06)	0.07
Adolescents’ gender^d^	1.384 (0.73-2.62)	0.18	1.419 (0.75-2.70)	0.19
Rule-setting	0.966 (0.63-1.48)	0.02	0.962 (0.63-1.47)	0.02
Adolescent involvement in rule-setting	1.060 (0.79-1.42)	0.03	–^e^	–
Rule-setting* involvement	1.023 (0.77-1.36)	0.01	–	–
Positive parenting	–	–	0.838 (0.59-1.20)	0.10
Rule-setting*positive parenting	–	–	1.044 (0.69-1.59)	0.02

a OR: odds ratio.

b CI: confidence interval.

c *d*: Cohen *d*

d Reference category: boy.

e Not available.

**Table 4.** Results of two-way interaction effects with internet-specific rule-setting in predicting the onset of at-risk/problematic social media use.

Variable	Model 2c		Model 2d	
	OR^a^ (95% CI)	*d^b^*	OR (95% CI)	*d*
Adolescents’ age	0.877 (0.72-1.07)	0.07	0.875 (0.72-1.07)	0.07
Adolescents’ gender^c^	1.484 (0.78-2.82)	0.22	1.487 (0.76-2.93)	0.22
Rule-setting	0.958 (0.62-1.49)	0.02	0.939 (0.60-1.46)	0.04
Phubbing	0.877 (0.72-1.07)	0.07	0.875 (0.72-1.07)	0.07
Rule-setting*phubbing	0.977 (0.72-1.33)	0.01	–^e^	–
Quality of coparenting^d^	1.246 (0.91-1.71)	0.12	–	–
Rule-setting*coparenting	–	–	0.877 (0.64-1.21)	0.07

a OR*: *odds ratio.

b *d*: Cohen *d.*

c Reference category: boy.

d For the analyses with quality of coparenting, adolescents from single-parent families (n=26) were excluded from the sample.

e Not available.

**Table 5.** Results of two-way interaction effects with internet-specific rule-setting in predicting the onset of at-risk or problematic social media use.

Variable	Model 2e		Model 2f	
	OR^a^ (95% CI)	*d* ^b^	OR (95% CI)	*d*
Adolescents’ age	0.556^c^ (0.333-0.93)	0.32	0.884 (0.00-0.22)	0.07
Adolescents’ gender^d^	1.395 (0.73-2.67)	0.18	1.407 (0.74-2.68)	0.19
Rule-setting	0.835 (0.53-1.30)	0.10	0.992 (0.66-1.58)	0.00
Rule-setting*age	2.171^e^ (1.35-3.49)	0.43	–^f^	–
Rule-setting*gender	–	–	0.945 (0.54-1.64)	0.03

a OR: odds ratio.

b *d*: Cohen *d*.

c Insignificant after applying Benjamini-Hochberg procedure.

d Reference category: boy.

e *P* ≤.001.

f Not available.

#### Three-Way Interactions

Models 3a-3h were performed to test whether the moderation effects of adolescent involvement in rule-setting, positive parenting, parental phubbing, and quality of coparenting on the association between internet-specific rule-setting and the onset of at-risk or problematic social media use depended on either adolescents’ age or gender. No significant 3-way interaction effects were found (Tables S1-S4 in Multimedia Appendix 1).

## Discussion

### Principal Findings

Concerned about problematic social media use, many parents apply restrictive mediation [1]. However, it remains unclear whether restrictive mediation is effective. This literature shows inconsistent associations between restrictive mediation and adolescents’ problematic social media use, underscoring the need to disentangle possible preventive effects from intervention effects and to investigate potential moderators. Therefore, in this study, we specifically focused on the preventive effect and took into account among whom, how, and under what conditions internet-specific rules were set. More specifically, we investigated the prospective relationship between rules about the amount, location, and timing of internet use and the onset of adolescents’ at-risk or problematic social media use. In addition, we examined the moderating role of demographic and parenting factors, including adolescents’ age, gender, adolescent involvement in rule-setting, positive parenting, parental phubbing, and quality of coparenting (2-way interactions). Furthermore, we tested whether the moderation effects of the parenting factors varied by adolescents’ age and gender (3-way interactions). Contrary to our expectations, we did not find a significant main effect of internet-specific rule-setting on the onset of at-risk or problematic social media use, nor any moderating effects of the parenting factors. However, the effect of parental rules regarding internet use was moderated by adolescents’ age. When parents set more rules, adolescents aged <12.31 years were less likely to develop at-risk or problematic social media use, while adolescents aged >15.70 years were more likely to develop at-risk/problematic social media use. No significant 3-way interactions were found.

In line with recent meta-analyses on restrictive mediation and problematic internet and media use [3,4], we found a moderation effect of adolescents’ age. For younger adolescents, parental rules as an external control seem beneficial to prevent at-risk and problematic social media use. However, from around age 12 years, when adolescents transition from primary to secondary school, the effectiveness seems to disappear. This is also in line with the cross-sectional study of Sanders et al [51] showing that restrictive mediation was related to less screen time in children up to 12 years, but not in children older than 12 years. This shift may be explained by the fact that, from this period, adolescents start placing greater importance on friendships and forming peer groups. As a result, peers may become more influential while parents may become less influential [52]. From the age of around 16 years onward, rules even seem to backfire, probably due to the intensifying need for independence [53,54]. As adolescents mature, their desire for autonomy intensifies, leading them to reject parental controls that they perceive as infringing on their freedom. This reaction is well explained by the psychological reactance theory, which posits that individuals experience a motivational arousal when they perceive that their freedom is threatened [55]. This reactance drives individuals to restore their lost autonomy, often by engaging in the restricted behavior, which makes the restricted behavior more appealing. For example, White et al [56] provided empirical support for this theory. In their study involving undergraduate students aged 18‐23 years, participants were asked to think back a few years and reflect on their secondary school years. The findings revealed that more restrictive mediation was related to more positive attitudes toward the restricted media content and more frequent viewing of the restricted content with friends [56]. In addition, older adolescents spend more time outside direct parental supervision [57,58], which gives them more opportunities to engage in social media use. Furthermore, a recent study showed that between the age of 14.55 and 17 years, more internet time rules were related to poorer quality of the parent-child relationship [59], and poorer parent-child relationship quality has consistently been linked to problematic social media use [5]. This could also explain why internet-specific rules seem to have the opposite effect at a later age in adolescence.

Our findings offer no support for the idea that the effect of internet-specific rules depends on how and under what conditions they are set. This is surprising, especially regarding the moderating effect of positive parenting and adolescent involvement in rule-setting, as previous research provides evidence for the role of autonomy as a necessary condition for effective parenting in relation to, for example, problematic gaming [60] and media violence exposure and aggression [61]. In addition, we found no evidence that these moderation effects depend on adolescents’ age or gender. These insignificant effects may reflect insufficient power to detect small effect sizes. Post hoc power analyses for logistic regression using G*Power revealed that our sample size of 315 participants provided 100% power to detect strong effects (OR 0.4/3), 90%‐95% power to detect medium effects (OR 0.7/1.5), but only 20%‐22% power to detect small effects (OR 0.9/1.1). Thus, although we cannot rule out the presence of any two- or three-way interaction effects of adolescents’ gender, involvement in rule-setting, positive parenting, parental phubbing, and quality of coparenting, it seems that such possible effects are unlikely to be medium or large. Future studies should consider including the enforcement of rules, as the impact of rules may be more influenced by the way they are enforced (eg, harsh disciplinary techniques such as punishment vs constructive techniques such as scaffolding with rational discussion of rules [24]) rather than the process and context in which they are established.

### Strengths and Limitations

One of the primary strengths of this study is that it is the first to describe the full nature of the interaction between rule-setting and adolescents’ age across all relevant levels of the moderator, providing information on the specific ages at which the significance and direction of the effect changed. Another strength is that we included the onset of at-risk and problematic social media use during the study as the outcome variable, allowing us to establish the potential preventive effect of internet-specific rule-setting. However, this strength came with the disadvantage that we had to remove adolescents who reported more than 2 symptoms in the first wave. In addition, the study was conducted over a period of 1.5 years, which means that more immediate or long-term effects may not have been captured. Moreover, low scores on positive parenting were rare, if not absent, in our study sample, which may have constrained our ability to detect a moderating effect. Furthermore, adolescent- and parent-reported data may be susceptible to response bias, such as social desirability or memory recall problems. For example, the assessment of adolescent involvement in rule-setting relied on a single item, and we used the average of both parents’ scores. This approach may have introduced bias, as parents might overestimate the degree of involvement, and the agreement between parents on the parent-reported variables was low. In addition, lower-educated families were underrepresented in this sample, limiting the generalizability of the findings. Finally, the study period coincided with the COVID-19 pandemic in the Netherlands, which significantly disrupted daily routines. These circumstances may have influenced the behavior of both parents and adolescents, possibly impacting the study’s findings.

### Implications

The implications of our findings are significant for both research and practical applications. From a scientific perspective, our results highlight the necessity for future studies to examine the effect of parenting practices on problematic social media use for early, middle, and late adolescence separately. Practically, our findings suggest that for children up to approximately 12 years, parents should be encouraged and advised to set rules about internet use, as this can help prevent the development of problematic social media use. However, as adolescents grow older, especially from the age of 16 years onward, parents should be advised to adopt more flexible and autonomy-supportive strategies to avoid resistance and negative outcomes. Especially since research has demonstrated that the way parents have an influence on adolescents’ behavior evolves during adolescence but does not vanish [62]. Parenting programs and interventions should incorporate age-specific guidelines and emphasize the importance of adapting parenting strategies as children grow older. These programs should educate parents about the different needs and responses of younger versus older adolescents to different parenting practices.

### Conclusions

Regardless of how and the conditions under which parents set internet-specific rules, these rules seem to have a protective effect for younger adolescents and the opposite effect for older adolescents. This highlights the need for studying parenting effects on social media use by age group and the importance of age-appropriate strategies in parental mediation. Understanding the changing needs of adolescents at different ages can help parents develop more effective strategies that support autonomy while also helping to prevent problematic social media use.

## Supplementary material

10.2196/64252 Multimedia Appendix 1 Tables showing the results of the 3-way interaction effects.

## Abbreviations

CHERRIES: Checklist for Reporting Results of Internet E-Surveys
OR: odds ratio
SMD: social media disorder
