# Supplementary material for: Parental Internet-Specific Rules and the Onset of Adolescents’ Problematic Social Media Use: Prospective Study Testing Potential Moderators
Source: J Med Internet Res. 2025 Sep 18;27:e64252. doi: 10.2196/64252 (PMC12445780; doi:10.2196/64252)
Supplement: Multimedia Appendix 1 [file jmir-v27-e64252-s001.docx]

**Multimedia Appendix 1**

**Table S1**

*Three-Way Interaction Effects With Adolescents’ Age in Predicting the Onset of At-Risk/Problematic Social Media Use*

| Variable | Model 3a | | | Model 3b | | |
| --- | --- | --- | --- | --- | --- | --- |
|  | *OR* | *95% CI* | *d* | *OR* | *95% CI* | *d* |
| Adolescents’ age | 0.534* | [0.311, 0.916] | 0.35 | 0.522* | [0.309, 0.879] | 0.36 |
| Adolescents’ gender^a^ | 1.371 | [0.706, 2.661] | 0.17 | 1.429 | [0.737, 2.772] | 0.20 |
| Rule-setting | 0.825 | [0.515, 1.320] | 0.11 | 0.806 | [0.516, 1.262] | 0.12 |
| Adolescent involvement in rule-setting | 1.107 | [0.766, 1.600] | 0.06 |  |  |  |
| Rule-setting*age | 2.261*** | [1.331, 3.842] | 0.45 | 2.236** | [1.366, 3.661] | 0.44 |
| Rule-setting*involvement | 0.695 | [0.444, 1.089] | 0.20 |  |  |  |
| Involvement*age | 0.694 | [0.441, 1.094] | 0.20 |  |  |  |
| Rule-setting*involvement*age | 0.972 | [0.674, 1.402] | 0.02 |  |  |  |
| Positive parenting |  |  |  | 0.784 | [0.516, 1.262] | 0.13 |
| Rule-setting*positive parenting |  |  |  | 0.976 | [0.573, 1.662] | 0.01 |
| Positive parenting*age |  |  |  | 0.848 | [0.490, 1.467] | 0.09 |
| Rule-setting*positive parenting*age |  |  |  | 0.878 | [0.474, 1.627] | 0.07 |

*Note*. *OR* = odds ratio; *CI* = confidence interval. *d* = Cohen’s d.

^a^ Reference category = boy.

****p* ≤ .001. ***p* ≤ .01. **p* ≤ .05.

**Table S2**

*Three-Way Interaction Effects With Adolescents’ Age in Predicting the Onset of At-Risk/Problematic Social Media Use*

| Variable | Model 3c | | | Model 3d | | |
| --- | --- | --- | --- | --- | --- | --- |
|  | *OR* | *95% CI* | *d* | *OR* | *95% CI* | *d* |
| Adolescents’ age | 0.525* | [0.306, 0.900] | 0.36 | 0.543* | [0.317, 0.932] | 0.34 |
| Adolescents’ gender^a^ | 1.512 | [0.786, 2.910] | 0.23 | 1.525 | [0.763, 3.045] | 0.23 |
| Rule-setting | 0.794 | [0.498, 1.266] | 0.13 | 0.772 | [0.478, 1.247] | 0.14 |
| Phubbing | 0.782 | [0.521, 1.176] | 0.14 |  |  |  |
| Rule-setting*age | 2.184*** | [1.353, 3.526] | 0.43 | 2.067** | [1.223, 3.494] | 0.40 |
| Rule-setting*phubbing |  |  |  |  |  |  |
| Phubbing*age |  |  |  |  |  |  |
| Rule-setting*phubbing *age | 0.731 | [0.396, 1.349] | 0.17 |  |  |  |
| Quality of co-parenting^b^ |  |  |  | 1.052 | [0.715, 1.547] | 0.03 |
| Rule-setting*co-parenting |  |  |  |  |  |  |
| Co-parenting*age |  |  |  |  |  |  |
| Rule-setting*co-parenting*age |  |  |  | 1.359 | [0.850, 2.174] | 0.17 |

*Note*. *OR* = odds ratio; *CI* = confidence interval. *d* = Cohen’s d.

^a^ Reference category = boy. ^b^ For the analyses with quality of co-parenting, adolescents from single-parent families (n = 26) were excluded from the sample.

****p* ≤ .001. ***p* ≤ .01. **p* ≤ .05.

**Table S3**

*Three-way interaction effects with adolescents’ age in predicting the onset of at-risk/problematic social media use*

| Variable | Model 3e | | | Model 3f | | |
| --- | --- | --- | --- | --- | --- | --- |
|  | *OR* | *95% CI* | *d* | *OR* | *95% CI* | *d* |
| Adolescents’ age | 0.880 | [0.722, 1.074] | 0.07 | 0.878 | [0.721, 1.068] | 0.07 |
| Adolescents’ gender^a^ | 1.427 | [0.749, 2.719] | 0.20 | 1.439 | [0.752, 2.752] | 0.20 |
| Rule-setting | 1.012 | [0.631, 1.623] | 0.01 | 0.986 | [0.620, 1.567] | 0.01 |
| Adolescent involvement in rule-setting | 0.815 | [0.469, 1.417] | 0.11 |  |  |  |
| Rule-setting*gender | 0.930 | [0.531, 1.629] | 0.04 | 0.980 | [0.566, 1.699] | 0.01 |
| Rule-setting*involvement | 1.281 | [0.788, 2.081] | 0.14 |  |  |  |
| Involvement*gender | 1.583 | [0.782, 3.205] | 0.25 |  |  |  |
| Rule-setting*involvement*gender | 0.783 | [0.434, 1.414] | 0.14 |  |  |  |
| Positive parenting |  |  |  | 1.057 | [0.537, 2.082] | 0.03 |
| Rule-setting*positive parenting |  |  |  | 0.909 | [0.442, 1.871] | 0.05 |
| Positive parenting*gender |  |  |  | 0.980 | [0.318, 1.603] | 0.01 |
| Rule-setting*positive parenting*gender |  |  |  | 1.186 | [0.494, 2.846] | 0.09 |

*Note*. *OR* = odds ratio; *CI* = confidence interval. *d* = Cohen’s d.

^a^ Reference category = boy.

****p* ≤ .001.

**Table S4**

*Three-Way Interaction Effects With Adolescents’ Age in Predicting the Onset of At-Risk/Problematic Social Media Use*

| Variable | Model 3g | | | Model 3h | | |
| --- | --- | --- | --- | --- | --- | --- |
|  | *OR* | *95% CI* | *d* | *OR* | *95% CI* | *d* |
| Adolescents’ age | 0.877 | [0.715, 1.075] | 0.07 | 0.877 | [0.720, 1.068] | 0.07 |
| Adolescents’ gender^a^ | 1.501 | [0.781, 2.883] | 0.22 | 1.524 | [0.769, 3.023] | 0.23 |
| Rule-setting | 0.981 | [0.607, 1.586] | 0.01 | 1.036 | [0.634, 1.693] | 0.02 |
| Phubbing | 0.884 | [0.561, 1.394] | 0.07 |  |  |  |
| Rule-setting*gender | 0.995 | [0.555, 1.783] | 0.00 | 0.819 | [0.448, 1.494] | 0.11 |
| Rule-setting*phubbing | 1.229 | [0.836, 1.808] | 0.11 |  |  |  |
| Phubbing*gender | 1.214 | [0.650, 2.265] | 0.12 |  |  |  |
| Rule-setting*phubbing *gender | 1.076 | [0.579, 2.000] | 0.04 |  |  |  |
| Quality of co-parenting |  |  |  | 0.971 | [0.624, 1.511] | 0.02 |
| Rule-setting*co-parenting |  |  |  | 0.761 | [0.529, 1.094] | 0.15 |
| Co-parenting*gender |  |  |  | 0.885 | [0.440, 1.778] | 0.07 |
| Rule-setting*co-parenting*gender |  |  |  | 1.223 | [0.658, 2.273] | 0.11 |

*Note*. *OR* = odds ratio; *CI* = confidence interval. *d* = Cohen’s d.

^a^ Reference category = boy.
